# A rare presentation of malignant mesothelioma of the tunica vaginalis managed with immunotherapy and review of the literature

**DOI:** 10.1002/ccr3.7610

**Published:** 2023-06-22

**Authors:** Kritika Mishra, Shihab Siddiquee, Anna Rachelle Mislang

**Affiliations:** ^1^ College of Medicine and Public Health Flinders University Bedford Park South Australia Australia; ^2^ Royal Adelaide Hospital Central Adelaide Local Health Network Adelaide South Australia Australia; ^3^ Faculty of Health and Medical Sciences University of Adelaide Adelaide South Australia Australia; ^4^ Clinpath Pathology Adelaide South Australia Australia; ^5^ Department of Medical Oncology, Flinders Centre for Innovation in Cancer Flinders Medical Centre Bedford Park South Australia Australia

**Keywords:** genomic profiling, orchiectomy, systemic immunotherapy, testicular mesothelioma

## Abstract

**Key Clinical Message:**

We describe the first case in literature of malignant mesothelioma of the tunica vaginalis that has shown partial response to systemic immunotherapy (ipilimumab‐nivolumab) post orchiectomy, warranting further investigation in a trial setting.

**Abstract:**

We present a case report of an 80‐year‐old ex‐smoker with a rare diagnosis of metastatic mesothelioma of the tunica vaginalis, managed with immunotherapy. The patient, with no known history of asbestos exposure, presented with a left scrotal mass and pain. Scrotal ultrasound confirmed a large paratesticular mass, and computed tomography (CT) of the chest, abdomen, and pelvis revealed a bilobed mass in the left scrotal compartment without associated inguinal or abdominopelvic lymphadenopathy, and an indeterminate, subcentimeter, bi‐basal subpleural nodules. He underwent a left orchiectomy, and histopathology confirmed the diagnosis of a paratesticular mesothelioma. Postoperatively, the patient had a positron emission tomography (PET) scan showing a new right pleural effusion as well as increasing size of the lobar and pleural nodules bilaterally, all metabolically active and suggestive of progressive metastatic disease. The patient was commenced on ipilimumab and nivolumab immunotherapy, a regimen indicated for malignant pleural mesothelioma; however, the efficacy on paratesticular mesothelioma is not known. After 6 months of treatment, the patient demonstrated a partial response to immunotherapy, with a reduction in the size of known pleural nodules and effusion.

Literature review suggests that diagnosis requires a high index of suspicion and patients commonly have metastatic disease at the time of diagnosis. Orchiectomy is a common management modality. However, the role, regimen, and benefits of systemic therapy are unclear, warranting further studies investigating management strategies.

## 1 INTRODUCTION

Malignant mesothelioma of the tunica vaginalis testis is a rare condition, constituting 0.3%–0.5% of all mesotheliomas.^1^, ^2^ This condition is most commonly diagnosed in men in their sixth decade of life.^3^ Mesothelioma is a tumor developing from mesothelial cells, which line the pleura, peritoneum, pericardium, and testis.^3^ Asbestos exposure is a well‐documented risk factor for the development of mesothelioma; however, is less commonly associated with mesothelioma of the tunica vaginalis.^4^ The tunica vaginalis is a serous membrane covering the anterior testis and epididymis, and a part of the peritoneum, which descends with the fetal testis from the abdomen into the scrotum.^5^ Due to the rarity of the condition and unknown pathogenesis, diagnosing malignant mesothelioma of the tunica vaginalis requires a high index of suspicion in patients presenting with common manifestations, such as a hydrocele.^1^ Diagnosis is predominantly achieved in a postsurgical setting through histopathology following orchiectomy.^4^ The majority of tunica vaginalis mesotheliomas are malignant and epithelioid in nature, while some patients have biphasic tumors.^3^ Most patients have aggressive disease, and up to 65% of patients die as a result of the diagnosis.^3^ There is no known standard of care for this disease, contributing to poor prognosis.^4^

## 2 CASE PRESENTATION

An 80‐year old fit male with an Eastern Cooperative Oncology Group (ECOG) performance status of 0 presented with left scrotal mass and pain, on the background of a 15 pack year smoking history, no known asbestos exposure and no history of scrotal trauma. His medical and surgical history were significant for prostate cancer in 2000, managed with prostatectomy and active surveillance, hypertension, and paroxysmal atrial fibrillation.

## 3 INVESTIGATIONS

**FIGURE 1:**
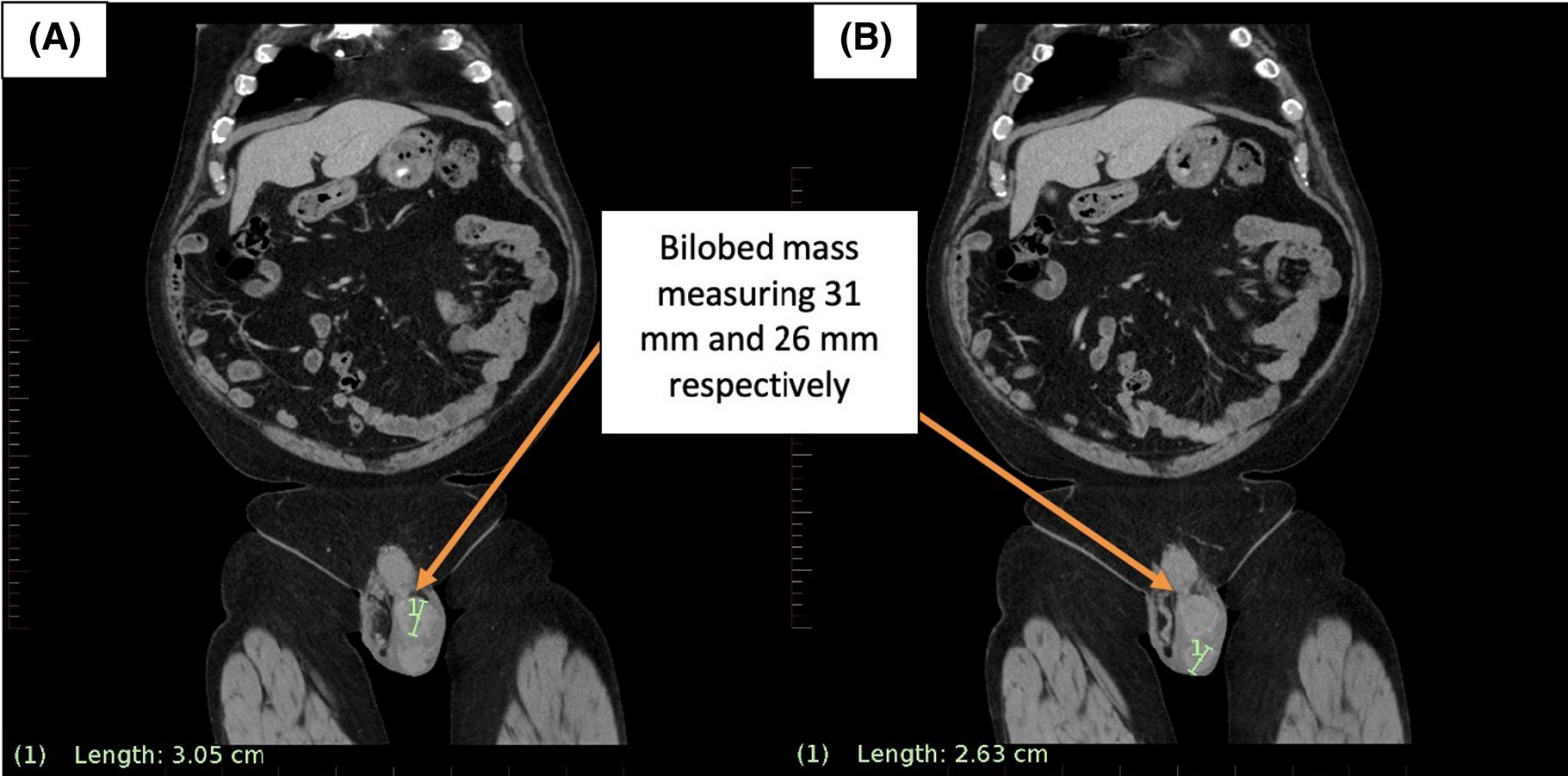
Pelvis CT demonstrating a bilobed mass compressing the left testicle, measuring 31 mm (A) and 26 mm (B) in size respectively, in keeping with ultrasonographic findings.

**FIGURE 2:**
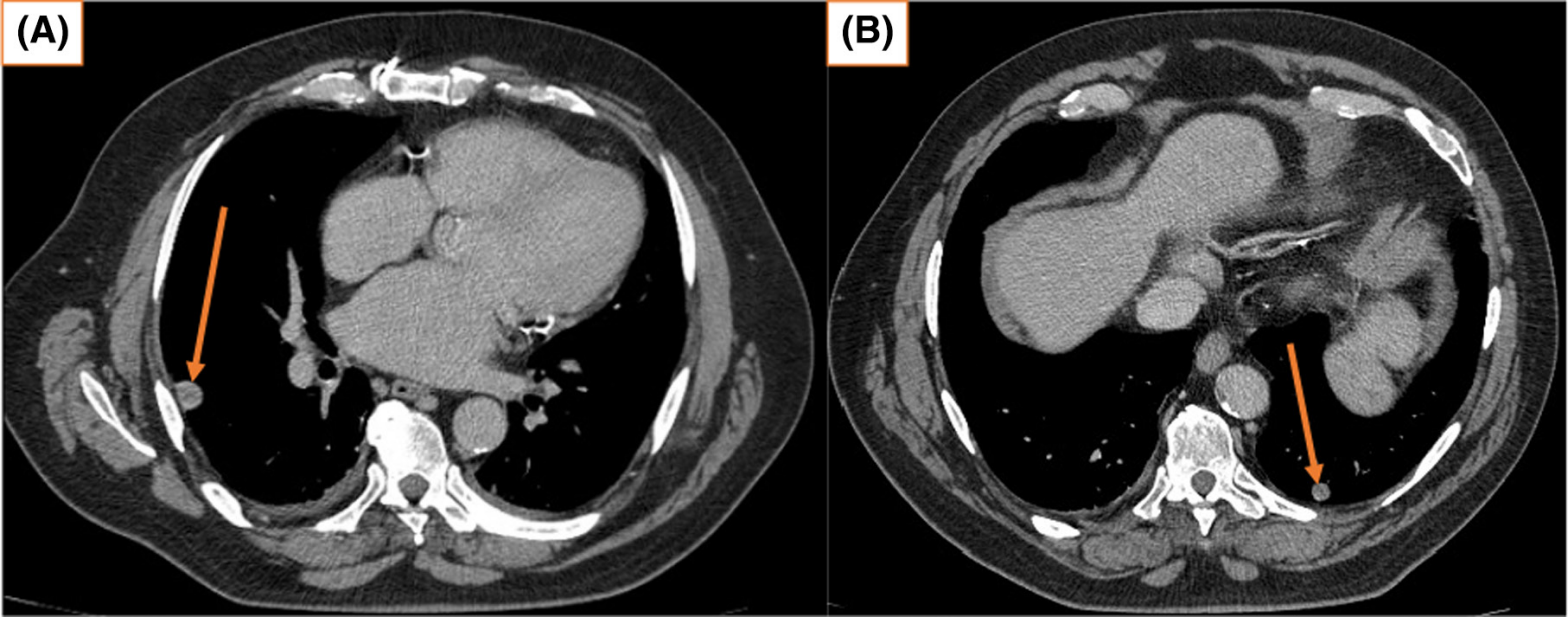
(A) May 2022, chest CT showing an indeterminate 13 mm pleural nodule in the right lower lobe, suggestive of right pleural metastasis (pre‐orchiectomy). (B) May 2022 chest CT showing a 9 mm pulmonary nodule in the left lower lobe, suggestive of left lung metastasis (pre‐orchiectomy).

Testicular tumor markers, including serum beta–human chorionic gonadotropin (β‐hCG), alpha–fetoprotein (AFP), and lactate dehydrogenase (LDH) were all within normal limits. Ultrasound examination of the left scrotum revealed a 66 × 26 × 38 mm mixed hyperechoic mass with increased vascularity and was suspicious for a neoplastic process. Computed tomography (CT) scan confirmed the sonographic findings, including a bilobed mass measuring 31 mm (Figure 1A) and  26 mm (Figure 1B) in diameter, and engorged vascularity in the left spermatic cord and left scrotal compartment. No retroperitoneal or inguinal lymphadenopathy was identified. CT scan of the chest found an indeterminate 13 mm pleural nodule in the right lower lobe and a 9 mm pulmonary nodule in the left lower lobe, equivocal for metastatic disease (Figure 2).

**FIGURE 3:**
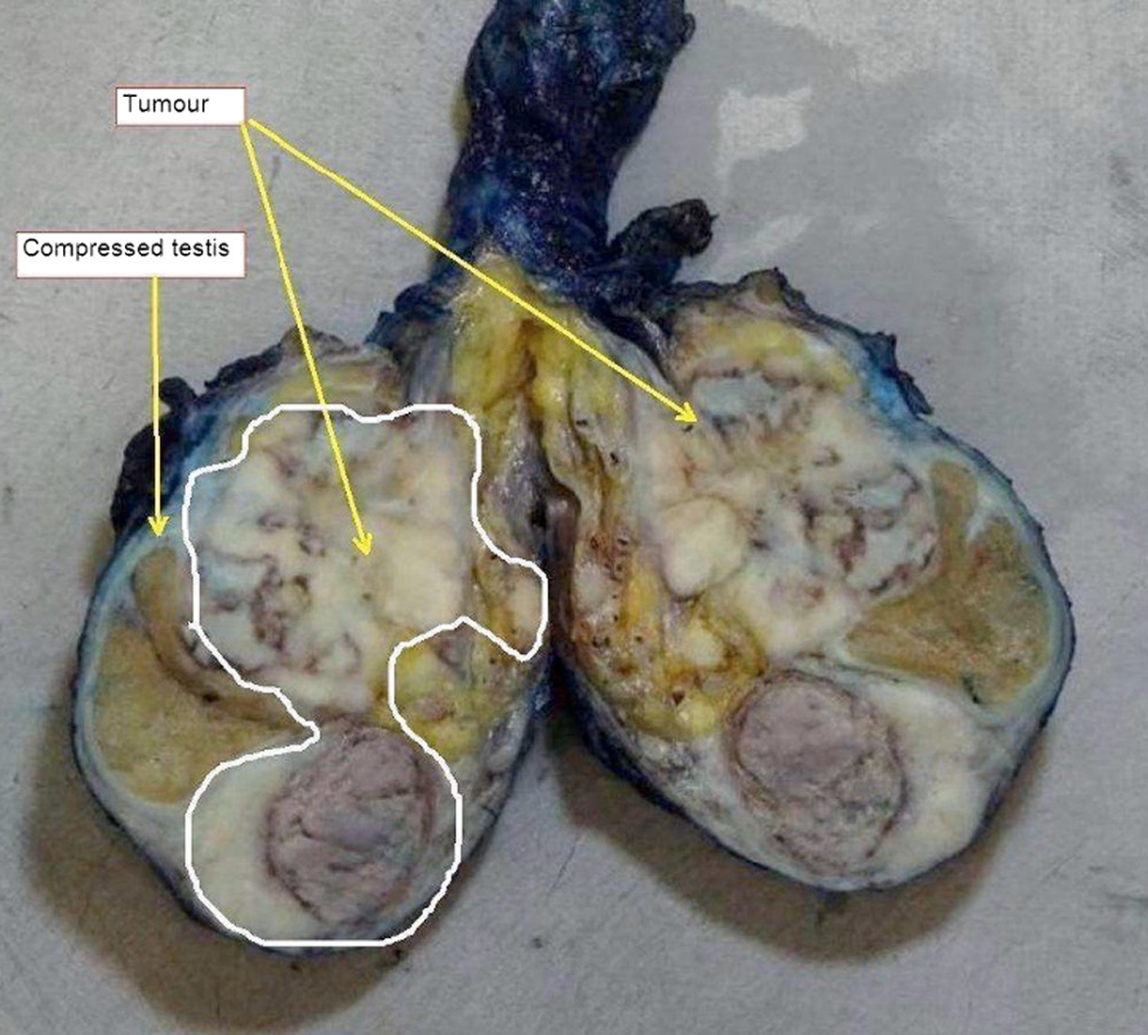
Macroscopic view of resected specimen illustrating a bilobed tumor compressing the testis, in keeping with radiological imaging.

**FIGURE 4:**
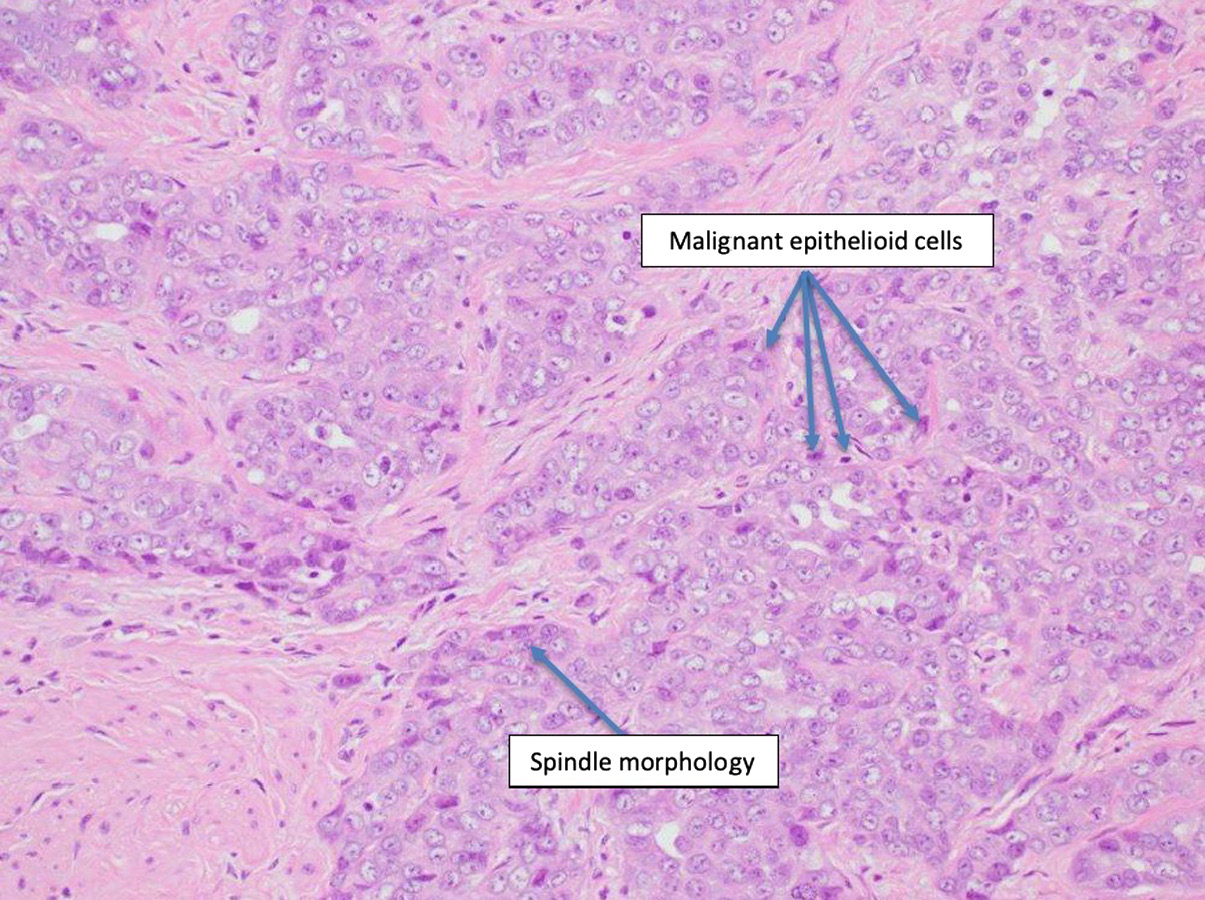
Microscopic view of pathological specimen diagnostic for malignant mesothelioma of the tunica vaginalis. Histology demonstrates infiltrative malignant epithelioid cells, extensive invasion and some areas of spindle morphology. A panel of immunohistochemical stains including calretinin confirm the diagnosis of mesothelioma.

Subsequently, the patient underwent a left orchiectomy. The macroscopic view of the resected specimen confirmed the presence of a bilobed tumor compressing the testicle (Figure 3). Histopathology and immunohistochemistry were diagnostic of mesothelioma of the tunica vaginalis (Figure 4).

**FIGURE 5:**
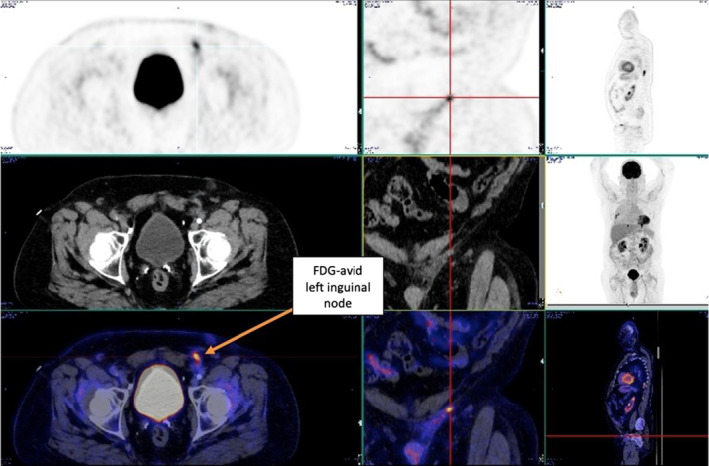
FDG‐avid residual left inguinal node post orchiectomy, suggestive of metastatic disease.

**FIGURE 6:**
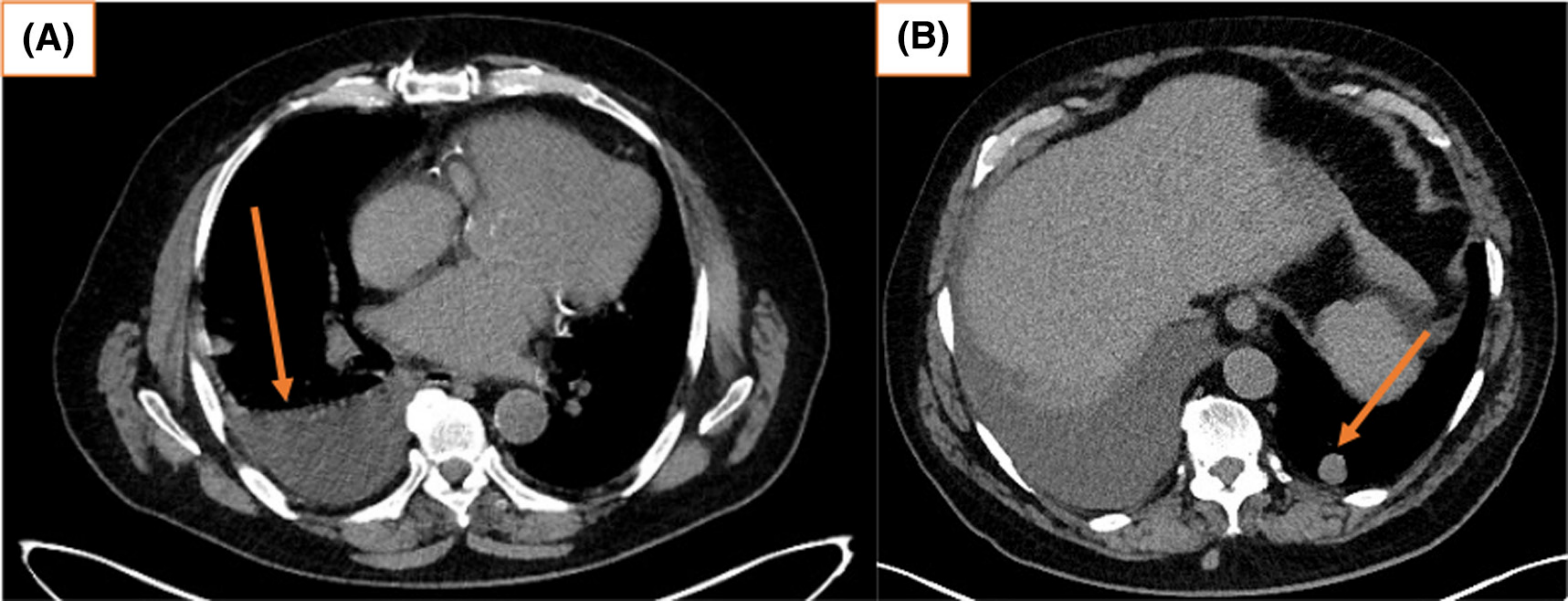
June 2022, chest CT immediately post–orchiectomy showing progression with new right pleural effusion (A) and enlarging left lung nodule (B).

Postoperatively, the patient underwent positron emission tomography (PET) with 358 MBq 18F‐FDG, and low dose CT scanning, confirming metastatic disease. Focal activity in the left inguinal region was suspicious of residual nodal malignant involvement (Figure 5). PET and CT post‐orchiectomy revealed a new right pleural effusion and enlargement of the left lung nodule (Figure 6), both of which were metabolically active on PET scanning, presumed metastatic and suggestive of disease progression. The patient was asymptomatic at the time of this imaging.

## 4 DIAGNOSIS

Based on these investigations, the large paratesticular mass was diagnosed as a malignant epithelioid mesothelioma of the tunica vaginalis with metastases to the left inguinal node, lung, and pleura.

## 5 TREATMENT

There is no standard systemic treatment for this rare cancer. However, given the progressive and metastatic nature of the disease and the patient's preference to have systemic treatment, he was offered ipilimumab and nivolumab immunotherapy. This occurred following discussions of potential immune‐related toxicity from a treatment regimen with unclear benefits due to lack of efficacy data among patients with mesothelioma to testis or tunica vaginalis, as these were excluded in the landmark trial CheckMate 743.^6^ There was no expenditure for the patient as drugs were accessed via the Pharmaceutical Benefits Scheme (PBS). Additionally, the patient was enrolled for prescreening in the Molecular Screening and Therapeutics (MoST) clinical trial and underwent molecular profiling to facilitate the identification of biomarkers or molecular targets for therapy. Genomic findings from a TruSight Oncology 500 panel revealed a microsatellite stable status and a low tumor mutational burden of 4.7 mut/Mb. However, no targetable mutation was found that meets the eligibility for current MoST studies. However, a frameshift truncating variant in tumor suppressor BAP1 (BAP1 V43Cfs*26) was identified, which could either be somatic or germline origin. Germline variants of BAP1 predispose to various cancer types, including malignant mesotheliomas; however, somatic variants are also frequently reported.^7^ Loss of functional BAP1 leads to deregulated cellular processes and may result in an altered therapeutic response to HDAC inhibitors.^8^ This patient has been referred to Clinical Genetics Unit for confirmatory germline testing.

## 6 OUTCOME AND FOLLOW‐UP

**FIGURE 7:**
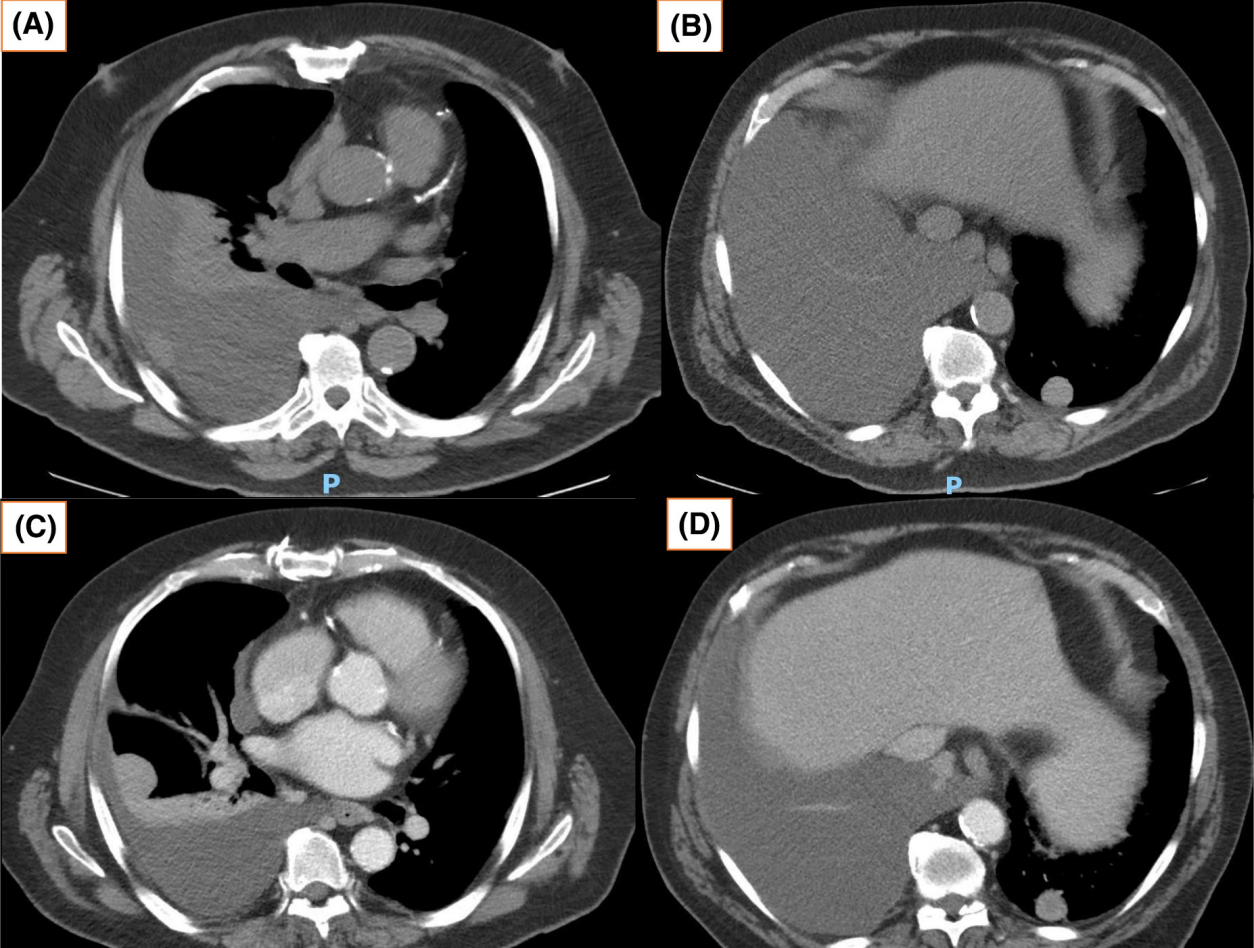
(A, B) July 2022, chest CT 1 week post C1D1 when the patient presented with increasing shortness of breath, demonstrating further disease progression. (C, D) July 2022 chest CT 1 week post drainage showing rapid accumulation of pleural effusion.

**FIGURE 8:**
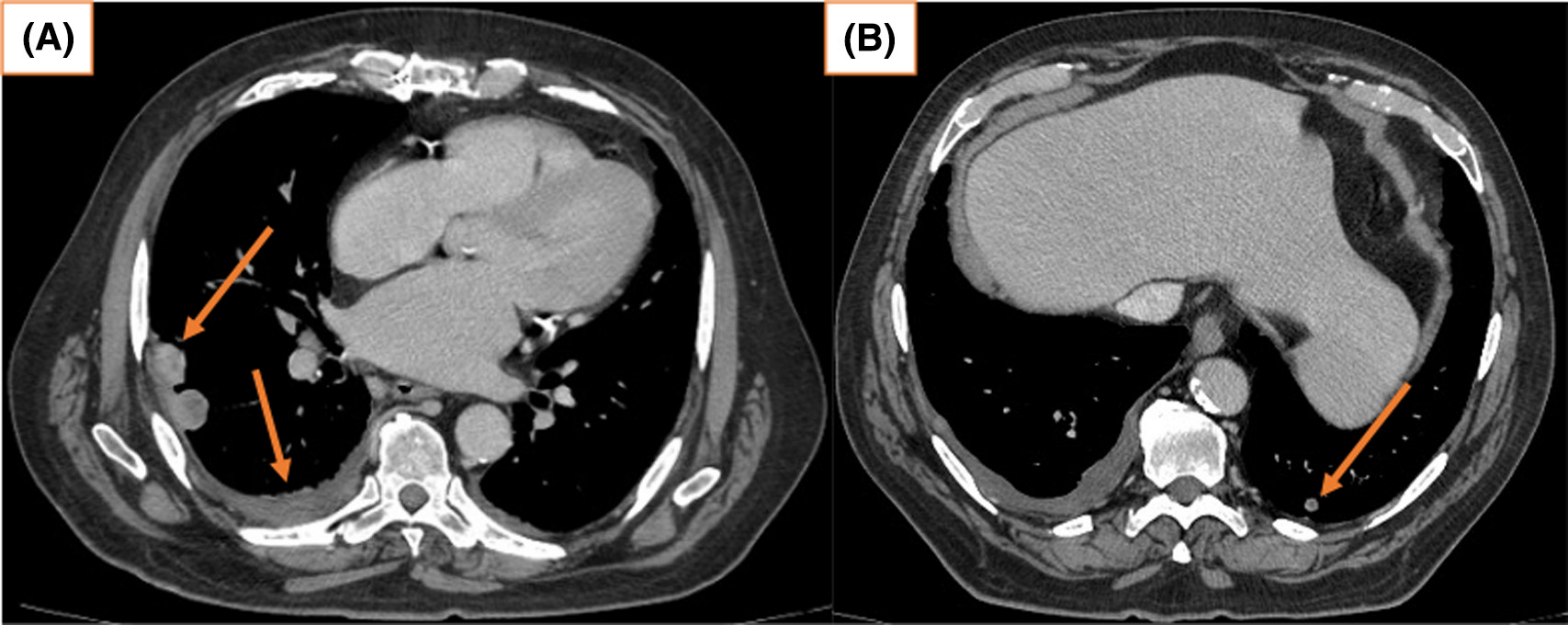
(A, B) October 2022, chest CT after 3 months of treatment showing partial treatment response with improvement in effusion without further thoracocentesis since discharge in July, and a reduction in the size of right pleural and left lung metastases.

**FIGURE 9:**
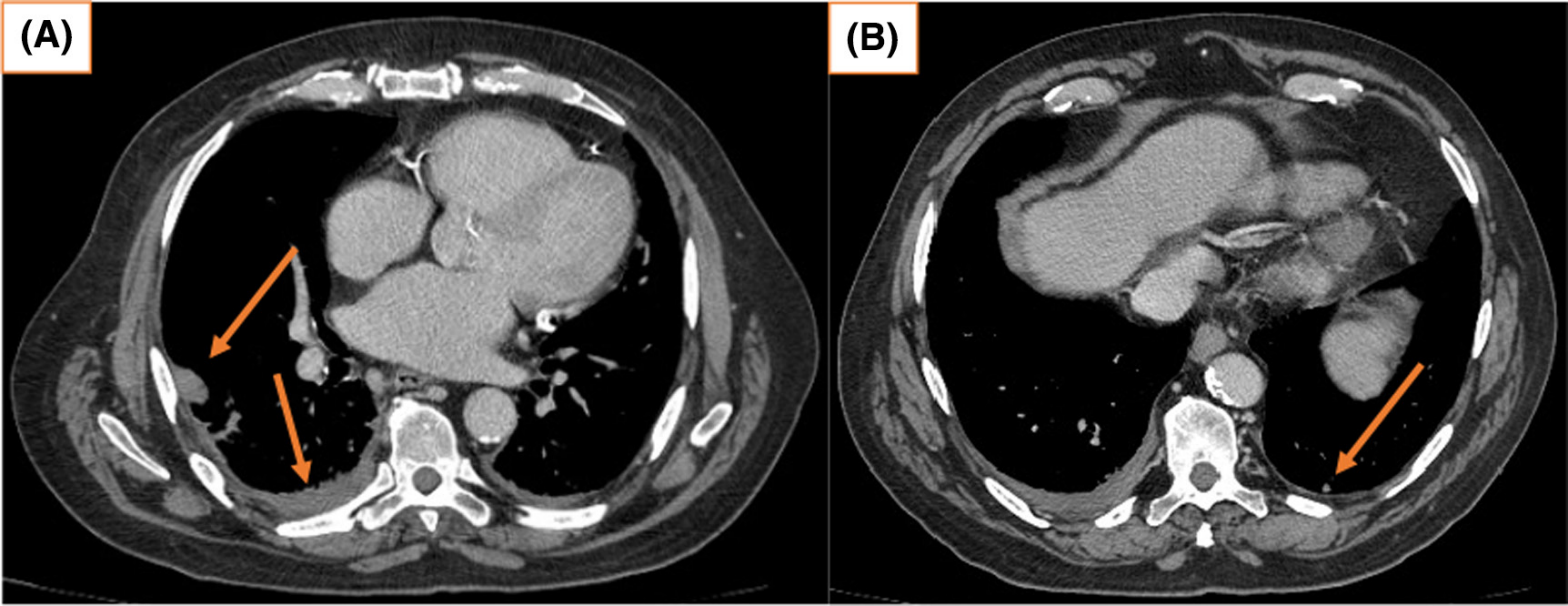
(A, B) January 2023, chest CT 6 months into systemic immunotherapy demonstrating ongoing partial response.

The patient presented to hospital with acute onset dyspnea and new oxygen requirement 1 week after cycle1 day1 (C1D1) of ipilimumab and nivolumab immunotherapy. CT scan of the chest demonstrated an increase in the size of the right‐sided pleural effusion since imaging one month ago (Figure 7), indicating a rapid disease progression. Diagnostic and therapeutic thoracocentesis and cytology confirmed malignant cells with features diagnostic of malignant mesothelioma, most likely representing pleural metastases. The patient was discharged home with rehabilitation, oncology, and respiratory follow‐up. Following 3 months of treatment, chest CT demonstrated a partial response to treatment, with improvement in effusion without further thoracocentesis and a reduction in the size of the right and left pleural metastases since discharge in July (Figure 8). At 6 months of treatment, the patient has demonstrated an ongoing partial response to immunotherapy with further reduction in the size of known pleural nodules and effusion (Figure 9). Adverse reactions include an immune‐mediated moderate (Grade 2) rash, managed with topical corticosteroids and 10 mg of oral prednisolone with good tolerance. The patient has not required further thoracocentesis and remains independent with his mobility and activities of daily living. As per the CheckMate 743 trial, the total duration of treatment is up to 2 years.

## 7 DISCUSSION

Malignant mesothelioma of the tunica vaginalis is a rare diagnosis and the management strategies are not standardized in clinical guidelines. A literature search on PubMed reveals that there are 119 papers published globally discussing this rare diagnosis. We reviewed 18 of these cases, diagnosed between 1994 and 2022, to highlight common risk factors, propensity for early, recurrent or metastatic disease, poor prognosis and lack of available standard treatment. Analysis of these 18 cases revealed that the mean age of diagnosis is 56 years (range: 14–81), and the disease most commonly manifests with scrotal swelling (89%) and ultrasound confirmed hydrocele (78%). Of the reported risk factors, in this patient cohort, one patient had a background of asbestos exposure, one patient had a smoking history and one patient had trauma history. Thus, our findings corroborate the literature review by Segura‐Gonzalez et al.,^4^ which describes that unlike mesotheliomas of the pleura and peritoneum, mesothelioma of the tunica vaginalis testis is less commonly associated with asbestos exposure, suggestive that other risk factors are involved in the pathogenesis of the tumor.^2^, ^4^, ^9^

When reported, testicular tumor markers were within the normal range for all patients. Ultrasound was the most common initial imaging modality, with 72% (13) of patients receiving sonography input. Of the other imaging modalities, 61% (11) of patients underwent a CT scan (pre or postoperatively), 17% (3) of patients received magnetic resonance imaging (MRI), and 17% (3) of patients underwent PET scans. Histopathology was diagnostic in all of cases. Immunohistochemical markers were reported in 15 cases, and of these there was positivity for calretinin (80%, *n* = 12), cytokeratin (67%, *n* = 10), vimentin (33%, *n* = 5), and epithelioid membrane antigen (20%, *n* = 3).

**FIGURE 10:**
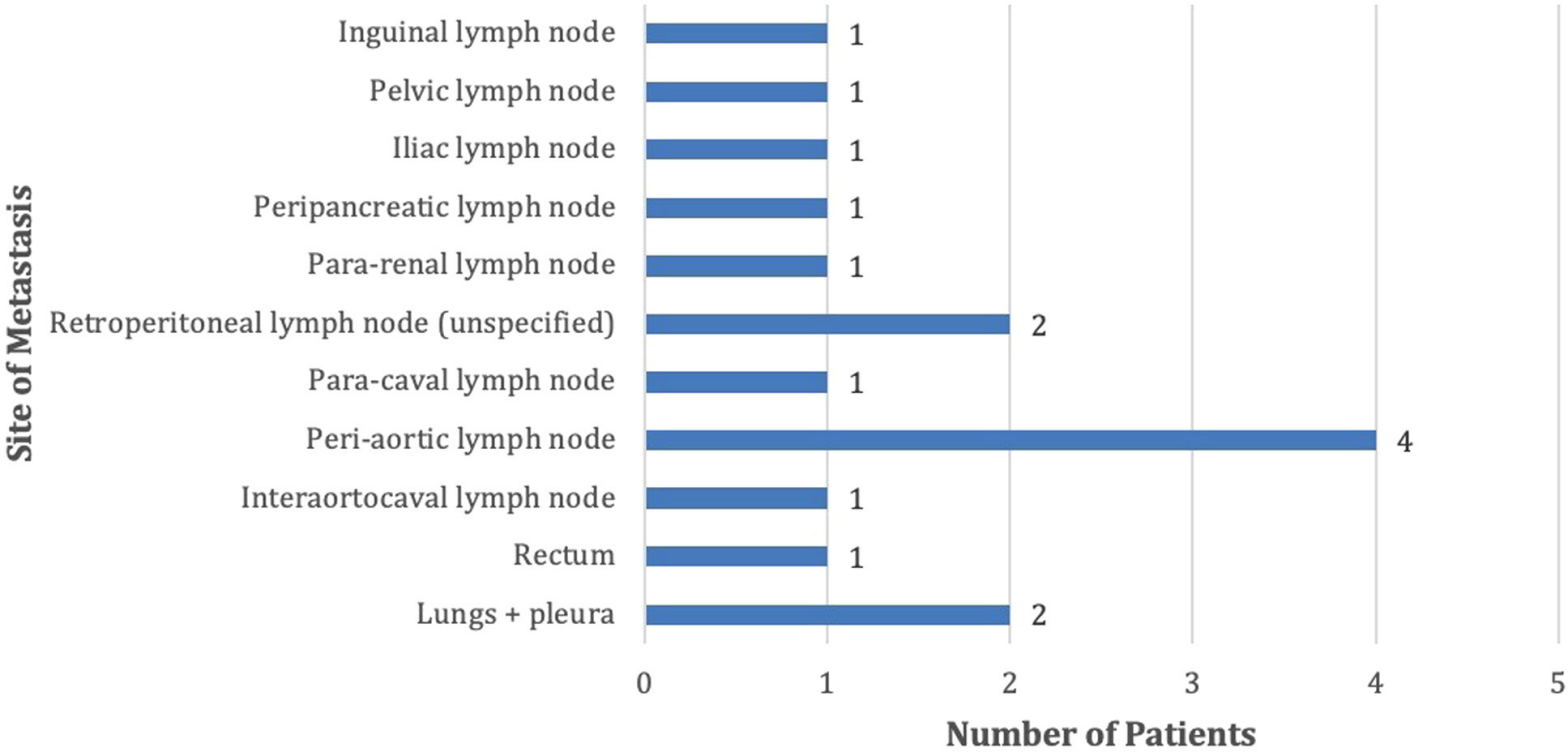
Site(s) of metastasis in patients with metastatic disease at the time of presentation (*n* = 8).

**FIGURE 11:**
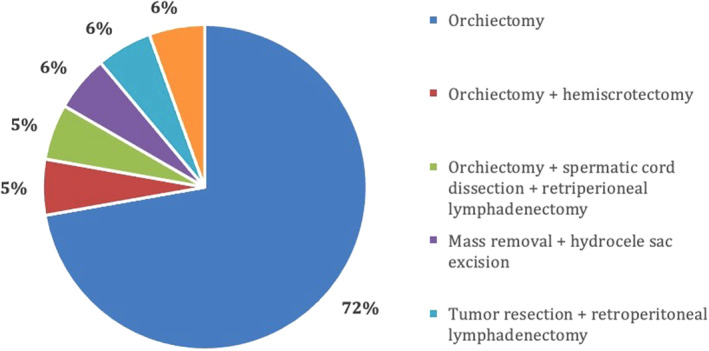
Initial surgical management of patients diagnosed with malignant mesothelioma of the tunica vaginalis testis.

At the time of diagnosis, 44% (8) of patients had metastatic disease, and the majority of patients had nodal involvement, two patients had lung metastasis (pulmonary and pleural) and one patient had rectal wall thickening (Figure 10). Due to the rarity of this diagnosis, there is no standard of therapy or guidelines for disease management. All patients were initially advised surgical management, with the most common surgical procedure being an orchiectomy, conducted in 83% of patients. The details of surgical management strategies in this patient cohort are illustrated in Figure 11.

Following surgery, some patients were managed with multimodal measures. Three patients underwent systemic therapy and the treatment regimen for all of these patients was cisplatin and pemetrexed. These chemotherapy regimens predated the CheckMate 743 trial, which concluded that immunotherapy with ipilimumab and nivolumab demonstrated better survival rates and decreased treatment‐related adverse events in comparison to cisplatin and pemetrexed.^6^ While the indications for systemic therapy were not documented in these case reports, one patient had disease invading the adjacent periorchium and spermatic cord, one patient had biphasic mesothelioma with lymph node involvement and one patient had mesothelioma with a minor biphasic component associated with worse prognosis.^3^ Two patients received chemoradiotherapy postoperatively, one of whom had locally advanced disease in the rectum and received gemcitabine and carboplatin and pelvis teletherapy,^4^ and one patient who had inter‐aortocaval and latero‐aortic lymph node dissemination received doxorubicin and cyclophosphamide with radiation to the left iliac chain and both paraaortic chains up to the level of the diaphragm.^10^

**FIGURE 12:**
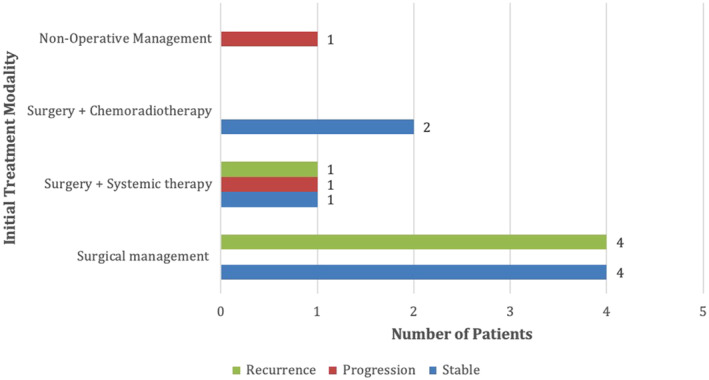
Disease patterns in patients with known prognosis (*n* = 14) receiving different treatment regimens, documented over 3 months–4.5 years.

Risk factors for poor disease outcome include older age at the time of diagnosis, tumor size greater than 49 mm, tumor necrosis, high mitotic index, angiolymphatic invasion, and biphasic cell type (epithelioid and sarcomatoid components).^3^, ^11^ The disease patterns of this patient cohort are documented over a three‐month to four–and–a–half–year time period (Figure 12). Forty‐four percent of the patients with disease progression and recurrence had metastatic disease at the time of diagnosis. Of those with known prognosis, 36% of patients developed recurrence, and 80% of these were within 3 years. Sixty‐seven percent of patients with metastatic disease at the time of diagnosis managed with surgery alone (*n* = 3) developed recurrence. The most common site of recurrence was nodal (mediastinal, retroperitoneal, aortic and inguinal), along with the skin and the lung.

Considering the rarity of this tumor, a multidisciplinary evaluation with medical, surgical, and radiation oncologists, along with urologists, should be utilized.^3^ Further studies are warranted to investigate surgical management options, and which systemic therapy regimen will improve clinical outcomes for patients.

## 8 LEARNING POINTS/TAKE HOME MESSAGES

1. In patients who present with a scrotal mass and/or hydrocele, malignant mesothelioma of the tunica vaginalis should be considered as a differential, even in the absence of documented risk factors.
2. Many patients have metastatic disease, most commonly nodal involvement, at the time of presentation.
3. There is no standard of treatment; however, commonly utilized management options include orchiectomy to remove the primary tumor, even in the setting of metastatic disease, plus or minus lymph node dissection.
4. The majority of cases recur after surgery. While patients may require systemic therapy, the treatment regimen is unknown.
5. We describe the first case of metastatic mesothelioma of the tunica vaginalis in literature which has shown partial response to systemic immunotherapy (ipilimumab‐nivolumab) post orchiectomy. This may pose a new treatment option for a rare disease and warrants further investigation in a trial setting.
6. Referral for genomic profiling may provide therapeutic or trial recommendations for this rare disease, and may even uncover a mutation of germline origin.

## AUTHOR CONTRIBUTIONS

**Kritika Mishra:** Conceptualization; data curation; formal analysis; investigation; methodology; writing – original draft; writing – review and editing. **Shihab Siddiquee:** Formal analysis; writing – review and editing. **Anna Rachelle Mislang:** Conceptualization; methodology; resources; supervision; writing – review and editing.

## ACKNOWLEDGMENT

Open access publishing facilitated by Flinders University, as part of the Wiley ‐ Flinders University agreement via the Council of Australian University Librarians.

## CONFLICT OF INTEREST STATEMENT

The authors declare no conflicts of interest.

## CONSENT

Written informed consent was obtained from the patient to publish this report in accordance with the journal's patient consent policy.

## Data Availability

The data that support the findings of this study are available from the corresponding author upon reasonable request.
